# VN-NDP: A Neighbor Discovery Protocol Based on Virtual Nodes in Mobile WSNs

**DOI:** 10.3390/s19214739

**Published:** 2019-10-31

**Authors:** Yuanyuan Zhang, Liangxiong Wei, Min Guo, Wei Wang, Yufang Sun, Junfeng Wang, Liangyin Chen

**Affiliations:** 1 School of Computer Science, Sichuan University, Chengdu 610065, China; yuanyuanzhang@stu.scu.edu.cn (Y.Z.); weilx_scu@aliyun.com (L.W.); guomin@stu.scu.edu.cn (M.G.); wang.david.wei@stu.scu.edu.cn (W.W.); chenliangyin@scu.edu.cn (L.C.); 2 School of Computer Engineering, Chengdu Technological University, Chengdu 610031, China; 3 School of Aeronautics and Astronautics, Sichuan University, Chengdu 610065, China; 4 Institute for Industrial Internet Research, Sichuan University, Chengdu 610065, China

**Keywords:** neighbor discovery, WSNs, energy-efficiency

## Abstract

As an indispensable part of Internet of Things (IoT), wireless sensor networks (WSNs) are more and more widely used with the rapid development of IoT. The neighbor discovery protocols are the premise of communication between nodes and networking in energy-limited self-organizing wireless networks, and play an important role in WSNs. Because the node energy is limited, neighbor discovery must operate in an energy-efficient manner, that is, under the condition of a given energy budget, the neighbor discovery performance should be as good as possible, such that the discovery latency would be as small as possible and the discovered neighbor percentage as large as possible. The indirect neighbor discovery mainly uses the information of the neighbors that have been found by a pairwise discovery method to more efficiently make a re-planning of the discovery wake-up schedules of the original pairwise neighbor discovery, thereby improving the discovery energy efficiency. The current indirect neighbor discovery methods are mainly divided into two categories: one involves removing the inefficient active slots in the original discovery wake-up schedules, and the other involves adding some efficient active slots. However, the two categories of methods have their own limitations. The former does not consider that this removal operation destroys the integrity of the original discovery wake-up schedules and hence the possibility of discovering new neighbors is reduced, which adversely affects the discovered neighbor percentage. For the latter category, there are still inefficient active slots that were not removed in the re-planned wake-up schedules. The motivation of this paper is to combine the advantages of these two types of indirect neighbor discovery methods, that is, to combine the addition of efficient active slots and the removal of inefficient active slots. To achieve this goal, this paper proposes, for the first time, the concept of virtual nodes in neighbor discovery to maximize the integrity of the original wake-up schedules and achieve the goals of adding efficient active slots and removing inefficient active slots. Specifically, a virtual node is a collaborative group that is formed by nodes within a small range. The nodes in a collaborative group share responsibility for the activating task of one member node, and the combination of these nodes’ wake-up schedules forms the full wake-up schedule of a node that only uses a pairwise method. In addition, this paper proposes a set of efficient group management mechanisms, and the key steps affecting energy efficiency are analyzed theoretically to obtain the energy-optimal parameters. The extended simulation experiments in multiple scenarios show that, compared with other methods, our neighbor discovery protocol based on virtual nodes (VN-NDP) has a significant improvement in average discovery delay and discovered neighbor percentage performance at a given energy budget. Compared with the typical indirect neighbor discovery algorithm EQS, a neighbor discovery with extended quorum system, our proposed VN-NDP method reduces the average discovery delay by up to 10.03% and increases the discovered neighbor percentage by up to 18.35%.

## 1. Introduction

In recent decades, thanks to the rapid development of electronic technologies and wireless communications, wireless sensor networks (WSNs) have attracted the attention of academics and industrialists [1,2]. The applications of WSNs are in a wide range of contexts, including but not limited to intelligent healthcare [3], military [4], environmental monitoring [4,5], smart city [6], disaster warning [6], agriculture [7], and industry field [8]. Furthermore, WSN is an indispensable part of Internet of Things (IoT), and the applications of WSNs are expanding fast with the blowout phenomenon of IoT in recent years [6]. A typical example of the application of IoT-oriented WSN is that WSNs can be used for collecting data in IoT in a large number of contexts, such as intelligent manufacturing sites. In general, a WSN is composed of many inexpensive micro sensor nodes and one sink node. These sensor nodes establish a network in a self-organizing manner. They collect data and forward the data to the sink node. The data collected by the sink node can be forwarded to the cloud since the sink node can usually access the Internet in a wired or wireless communication manner [9].

One major drawback of WSNs is the limited energy resources of sensor nodes; they are typically powered by batteries, and these batteries usually cannot be replaced or recharged [10]. As a result, the objective of prolonging the lifetime of the network mainly focuses on efficiently managing the energy resources throughout the entire network [11]. Neighbor discovery, the bootstrapping procedure of WSNs, is one of the fundamental functionality for many basic networking protocols, such as medium access control and routing [12]. Neighbor discovery is an energy-intensive operation; therefore, efficient energy management of the process is also vital. The reasons why neighbor discovery is energy-intensive are explained below. On one hand, neighbor discovery operation should continuously run throughout the network lifetime, since neighbor relationships may change because of deployment of new sensors [13] or sensors’ mobility in mobile WSNs [10]. On the other hand, it is necessary for a sensor to broadcast signals to other sensors if the sensor wants to be discovered by others, but wireless communication via the radio frequency (RF) module is power-wasting. For the purpose of energy conservation, it is desirable to let the node work in low duty cycle (DC) mode [14]. This means that the hardware is allowed to be in sleep mode most of the time and to wake up only if packets need to be exchanged. The term DC refers to the fraction of time during which a sensor node is in the active state. For example, a device whose DC is 2% is active during one time slot out of every 50 slots. Despite its efficiency in energy conservation, DC mode makes it difficult for the design of neighbor discovery protocols to limit neighbor discovery delay. Particularly, the two important design objectives, energy conservation via a duty-cycled operation mode and optimizing other neighbor discovery performances (i.e., minimizing discovery delay or maximizing discovered neighbor percentage), are contradictory to each other. Therefore, the main objective of designing duty-cycle based neighbor discovery protocols is to improve energy efficiency, that is, optimize energy consumption and other discovery performances. We can assess the energy efficiency of different neighbor discovery protocols by keeping energy consumption constant and comparing other aspects, i.e., discovery delay or discovered neighbor percentage. In addition, since clock synchronization is difficult in a distributed network, neighbor discovery must be done asynchronously. The requirement of asynchronism aggravates the challenge of devising energy-efficient neighbor discovery protocols.

Many asynchronous neighbor discovery protocols have been proposed [12] to improve energy efficiency in the last decade. These protocols are divided into two categories, namely direct methods and indirect methods [9]. Direct methods, also known as pairwise methods, are further divided into probabilistic and deterministic methods [15]. In probabilistic methods, sensor nodes’ working state (i.e., active or sleep state) follows a probability distribution with respect to time. In deterministic methods, sensor nodes switch between active and sleep states according to a pre-designed wake-up schedule which guarantees that a pair of neighbor nodes are activated simultaneously within a limited delay time, called worst-case bound discovery latency.

In order to further improve the energy efficiency of neighbor discovery methods in mobile WSNs, some indirect neighbor discovery methods were proposed [9,10,15,16,17]. Indirect discovery methods generally work with a pairwise method. They improve energy efficiency mainly by re-planning the states of some slots, that is, switching some sleep slots in the pairwise method to active slots or switching some active slots to sleep slots. The core idea of one category of indirect neighbor discovery methods is that a node can add a small number of active slots to discover potential neighbors through taking advantage of neighbor information provided by discovered neighbors. In the other category of indirect methods, the node can remove low-efficiency active slots whose control rights are originally owned by the underlying pairwise method. Although existing indirect methods achieve high energy-efficient performance, they have some limitations, and these limitations hinder further improvement of energy-efficiency. Regarding the methods that involve adding additional active slots, the main limitation is that some low-efficiency active slots still exist. For the methods involving the removal of low-efficiency active slots, although they are energy efficient in terms of maintaining neighborship between discovered neighbors, the main limitation is that the opportunity of discovering new neighbors is reduced. Possibly, there exist new neighbors at any moment in mobile WSNs. Some opportunities of discovering new neighbors are lost because a number of active slots are removed; hence, the well-designed wake-up schedules for guaranteeing deterministic discovery are destroyed. In this paper, we propose a virtual-node based indirect neighbor discovery protocol (VN-NDP) to address these limitations. One of the main highlights of the VN-NDP is that lower energy-efficient active slots are removed, and the integrality, or non-destructive quality, of wake-up schedules is guaranteed. As a result, new neighbors can be also discovered in a very high energy-efficient manner. The core ideas of VN-NDP are as follows:

- Sensor nodes that have discovered each other form a group in a cooperative way if the distance between any two nodes in the group is not more than a very small threshold value.
- The nodes in the group are regarded as a virtual-node. These member nodes share responsibility for the activating task of one node’s wake-up schedule and these nodes remain sleep in remaining slots. In other words, the combination of these nodes’ wake-up schedules forms the complete wake-up schedule of a node that only uses a pairwise method. In line with the principle of neighbors’ spatial similarity [9,10], the nodes with smaller spatial distance have more common neighbors. Therefore, the nodes in a group can share their discovered neighbors because the distance between any two nodes in the group is very small.

The main contributions of this paper are as follows:

- To the best of our knowledge, the concept of virtual-node in neighbor discovery protocols is introduced here for the first time. A virtual-node is a group of nodes whose group distance is less than a given threshold value; group distance is the maximal distance between any two nodes in the group. Note that the distance between two nodes is obtained based on the received signal strength indication (RSSI) signals received.
- We propose a set of mechanisms for group management throughout the group’s lifetime, including forming the group, cooperating operation in the group, and dismissing the group. In group management, we quantitatively analyze the operation that has a non-negligible impacts on energy efficiency; the optimal values of all mentioned parameters are deduced.

The rest of the paper is structured as follows: in Section 2, the main neighbor discovery algorithms in open literature are briefly introduced. In Section 3, we present the motivation of our proposed method in detail. Section 4 illustrates the design of our proposed method, especially group management mechanisms. The details of theoretical analysis are presented in Section 5. In Section 6, simulations and comparisons with other methods are presented. Finally, Section 7 concludes this paper and gives our research directions for future works.

## 2. Related Work

In self-organizing sensor networks, there is no precise synchronization clock between nodes in order to meet the requirements of low cost and low power consumption. Therefore, most neighbor discovery studies in self-organizing sensor networks are asynchronous [12,14]. In the past decade or thereabout, with the rapid development of self-organizing sensor networks, a large number of asynchronous neighbor discovery studies have emerged. The research methods used in these studies can be classified using different criteria. Currently, scholars are accustomed to using hierarchical classification methods. In the upper layer, neighbor discovery methods are divided into direct methods and indirect methods [9,12,15]. Direct methods are also called pairwise methods, and indirect methods are also called collaborative methods. In direct neighbor methods, node A is supposed to find node B only when node A receives a beaconing packet from B. In general, there are two layers in the indirect neighbor discovery method. The bottom one corresponds to the direct method, while the upper one is the indirect method. The function of the indirect neighbor discovery method in the upper layer is to use the public neighbor information obtained by the direct neighbor method in the bottom layer to efficiently re-plan the nodes’ work schedule [12]. Direct discovery methods are divided into probabilistic methods and deterministic methods depending on whether the nodes’ work schedules are determined in advance. Among the two methods above, the probabilistic ones generally have a low average discovery delay, but there is no guarantee that all neighbors can be found within a certain time. The deterministic ones ensure that there is a limit of discovery delay (also known as worst-case bound discovery latency), that is, a node must be able to discover all its neighbors within the worst-case bound discovery latency. Next, the above three categories of methods, namely the probabilistic methods, the deterministic methods, and the indirect methods, are reviewed separately.

In probabilistic methods, each node’s state, active or sleep state, in a certain time slot or time period is dependent on a certain probability. We briefly describe three typical probabilistic neighbor discovery methods, namely Birthday [18], PSBA (a prime-set-based neighbour discovery algorithm) [19], and Panda [20]. In the Birthday algorithm, time is divided into multiple time slots, and the working state of the node in each time slot, active or sleep state, depends on a certain probability. The PSBA selects the prime numbers of set *I* according to the DC required by the application; it randomly selects a prime number from *I* as the current wake-up period. After repeating this prime number α times, another prime number is randomly selected from *I* as the next wake-up period, and the loop is performed. PSBA combines the advantages of probabilistic and deterministic discovery protocols, which greatly reduces the delay of probabilistic algorithms. In the Panda method, nodes are switched between sleep, broadcast, and listening states. The sleep time is in accordance with the exponential probability distribution. The parameters in the Panda method are analyzed and optimized using stochastic process theory.

The deterministic neighbor discovery methods can be divided into equal-length and unequal-length time slot methods according to the time slot model used [21]. Typical equal-length time slot methods include the Quorum-based methods [22,23,24], the Prime-based methods [25,26], the Block-based method [13,27], the Searchlight [14], and the Diff-Code [28]. In Quorum-based methods, $m^{2}$ consecutive time slots form a two-dimensional array, and the node can randomly select one row and one column to be active. Disco and U-connect are prime-based deterministic methods. In the former method, according to the Chinese remainder theorem, each node selects two prime numbers $p_{1}$ and $p_{2}$, and the node is active in multiple time slots of each prime number. This active pattern guarantees that the worst-case bound discovery latency does not exceed $p_{1}p_{2}$. In U-connect, $p^{2}$ consecutive time slots are treated as a two-dimensional array, and the nodes are active in the first column; in addition, the nodes are active in the first half of the first line of the two-dimensional array, and this active pattern guarantees that the worst-case discovery delay does not exceed $p^{2}$. In the Block-based method, authors hold that the neighbor discovery problem is essentially equivalent to the shift and self-intersection of a set. Thus, according to the difference set and multiplier theorem in block theory, a feasible optimal active scheduling model is provided. The core idea of the Searchlight is that *t* time slots are composed of one period, and each period contains two active slots, namely, slots A and P. A is the first slot of each period. Since the period lengths are equal, the relative offsets of the A slots of the two nodes remain unchanged, and slot P is used to detect other nodes’ slot A for neighbor discovery. Diff-Code is so far the best asynchronous and deterministic discovery method using equal-length slots. In the Diff-Code method, the active-sleep pattern of node is formulated as a 0–1 code. Using set theory, a Diff-Code is obtained. The theoretical analysis result indicates that a Diff-Code is optimal when it can be extended from a perfect difference set.

The design motivation of unequal-length slot methods is that there is redundancy of slot overlap for pairwise deterministic algorithms with an equal-length slot regardless of whether the slots are aligned [14,21]. When the time slots are aligned, there is at least one complete active slot overlap within the worst-case delay. When the time slots are not aligned, there are at least two active overlaps within the worst-case delay, and the total length of the two overlaps is one time slot. In fact, it is sufficient to ensure that the overlap length of the active slot is slightly greater than the time δ, which is enough to transmit or receive one packet of data. In order to eliminate redundant overlapping time slots, numerous unequal-length time slot neighbor discovery methods have been proposed, including the equal-length active time slot methods of Searchlight-Striped [14] and Non-Integer [29], the unequal-length method of Lightning [21], and the dedicated sending/receiving time slot methods of Griassdi [30], G-Nihao [31], and THL2H (Talk Half Listen-2-Half) [32]. In the above methods, Searchlight-Striped converts the original Searchlight’s active time slot into (1+δ)ζ, where ζ is the length of a normal time slot. Non-Integer trims the slot length of many typical deterministic neighbor discovery protocols to (0.5+δ)ζ. Lightning improves energy efficiency by enhancing the P slots of the original Searchlight. Specifically, the P slot is replaced by a C slot, and the C slot includes two parts, IP (Idle Part) and BP (Beacon Part); only BP, whose length is δζ, is in an active state. In Griassdi, each node contains specialized listening slots and dedicated transmitting slots. The main idea of Griassdi is explained as below. $t_{L}$= $t_{S}-1$, where $t_{L}$ is the period of the listening slot and $t_{S}$ is the period of the transmission slot. Thus, the offset of the listening slot of one node A and the offset of the transmission slot of another node B are incremented by 1 after each period $t_{L}$. Therefore, after $t_{L}$ periods, there must be an offset between the listening slot of A and the transmission slot of B is 0, which means that A finds B. Node B can also find A in a similar way. G-Nihao can achieve shorter discovery latencies than most slotted protocols by sending additional beacons in each period. THL2H is an improved version of G-Nihao, and it divides the listening row from G-Nihao into two listening halves.

In addition, there are other discovery methods, such as self-adapting discovery methods [33,34] and the methods considering collision problems [33,35].

Based on the addition or removal of active slots of the original wake-up schedules in the underlying direct method, the indirect neighbor methods can be divided into the methods of increasing and reducing the active time slot. The indirect methods of increasing the active time slot include ACC (an on-demand generic discovery accelerating middleware) [16], Group-based [10], GBFA (Group-based Fast Algorithm) [9], SPND (a low-energy selectively proactive neighbor discovery) [36] and GPND (a general and probabilistic neighbor discovery middleware) [15]. In addition, the typical indirect method for reducing the active slot is EQS (a neighbor discovery with extended quorum system) [17]. In ACC, the slot gain is calculated for each active slot to be added, and the slot gain is obtained by temporal and spatial similarities. After the calculation, the *k* time slots with the largest slot gain are selected as the active slots to be added. In Group-based, a small number of active slots are added to verify whether the potential neighbors are true neighbors, and the number of public neighbors is the basis for adding additional time slots. GBFA optimizes the selection efficiency of adding additional active time slots on the basis of Group-based methods. In a dynamic scenario, neighbor relationships between nodes change frequently. In the SPND algorithm, the node can combine the movement model of the nodes in the network with the division of the set, selectively wake up at the moment when the neighbor wakes up, and confirm with the neighbor node. In order to facilitate the evaluation of neighbor relationships with multi-source neighbor information, GPND was proposed to represent neighbor relationships with continuous probability values between 1 and 0, rather than the discrete values of 1 and 0. Using this probability value, GPND improves the selection efficiency of the verifying slot in Group-based algorithms. On the other hand, the EQS protocol based on the Extended Quorum graph theory uses indirect discovery to further reduce energy consumption. Nodes in EQS can exploit their direct neighbor nodes to enhance their neighbor discovery process, thus some unnecessary active slots can be filtered out without affecting neighbor discovery.

Based on the in-depth investigation of existing neighbor discovery methods, especially indirect discovery methods, VN-NDP is proposed in this paper. VN-NDP combines the respective advantages of two categories of indirect methods, that is, not only adding a small number of additional efficient active slots, but also removing inefficient active slots. Therefore, VN-NDP greatly improves the efficiency of neighbor discovery. In addition, VN-NDP uses virtual nodes to achieve the goal of maintaining the integrity of the original nodes’ working schedule, thereby overcoming the main limitation of the EQS method. The limitation is that the possibility of discovering new neighbors is greatly reduced by removing some active time slots.

## 3. Motivation

Since our work is mainly motivated by EQS, firstly, we give a simple example to illustrate the key thoughts of EQS and its main limitations. Secondly, we present the motivation behind our method, VN-NDP, and describe how to use it to improve this example.

**Figure 1:**
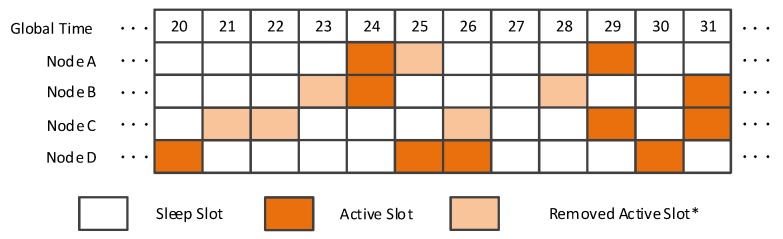
EQS when the pairwise method is Disco. EQS: A neighbor discovery with extended quorum system. (*A removed active slot means a sleep slot that is an active slot in the original wake-up schedule).

Similar to other indirect neighbor discovery methods, EQS works with a pairwise method and re-plans the working states of some slots in the wake-up schedule of the pairwise method used. Specifically, EQS uses a quorum graph to plan all possible neighbor discovery paths. By reducing redundant paths, some unnecessary active slots can be filtered out significantly. To some extent, using an EQS method can reduce energy consumption. In the example below, the pairwise method used is Disco. Assume that nodes A, B, C, and D are four nodes in a mobile network, and nodes A, B, and C all lie in the communication range of the other 2 and have found each other before global slot number 20 using the pairwise method Disco (see Figure 1). Assume that the working start time of nodes A, B, and C are global slots 4, 3, and 1, respectively. According to the pairwise method Disco, we can obtain the active slots of nodes A, B, and C from global slot 20 to global slot 31. Node A’s active slots from global slot 20 to global slot 31 are global slots 24, 25, and 29. Node B’s active slots from global slot 20 to global slot 31 are global slots 23, 24, 28, and 31. Node C’s active slots from global slot 20 to global slot 31 are global slots 21, 22, 26, 29, and 31. Applying the EQS method to the wake-up schedules of A, B, and C from global slot 20 to global slot 31, that is, filtering out lower energy-efficient active slots in this slot range, global slot 25 in node A, global slots 23 and 28 in node B, as well as global slots 21, 22, and 26 in node C are removed based on Extended Quorum theory. The remaining active slots, i.e., global slots 24 and 29 of node A, global slots 24 and 31 of node B, and global slots 29 and 31 of node C, can guarantee that nodes A, B, and C can discover each other before global slot 31. Next, we consider the case that node D gradually moves towards nodes A, B, and C and becomes neighbors of nodes A, B, and C in global slot 20. Unfortunately, node D fails to discover nodes A, B, and C before global slot 31 because some active slots of nodes A, B, and C are removed in the EQS method. Therefore, although the EQS method is highly energy-efficient in maintaining neighborship between discovered neighbors, such as neighbors B and C of node A, this method still incorrectly removes some active slots; therefore, some new neighbors may not be discovered.

**Figure 2:**
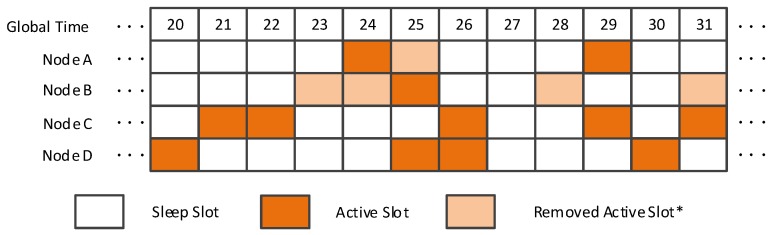
VN-NDP when the pairwise method is Disco.VN-NDP: A neighbor discovery protocol based on virtual nodes. (*A removed active slot means a sleep slot that is an active slot in the original wake-up schedule).

To address the limitation of an EQS method, that is, removing lower energy-efficient active slots but ensuring the discovery of new neighbors, our method, VN-NDP, is proposed. Figure 2 shows the arrangement of active slots from global slot 20 to 31 for nodes A, B, and C with the VN-NDP method, respectively. Since the nodes with smaller spatial distance between them have more common neighbors, they can join a group in VN-NDP if the distance between any two nodes is not more than a distance threshold value. In this example, the threshold value is set as 20 m. Since the distance between nodes A and B, B and C, and A and C are 15 m, 25 m, 30 m, respectively, only A and B can form a cooperative group; thus, nodes A and B are regarded as a virtual node. From global slot 20 to 31, nodes A and B share responsibility for the activating task of one node, i.e., node A. Note that, since our VN-NDP is an indirect neighbor discovery method combined with a paired method, the DCs of the nodes in the group are the same. We can randomly select a node’s wake plan as the work plan of the virtual group. Therefore, the active slots of node A from slot 20 to 31 are slots 24 and 29, and the active slot of node B from slot 20 to 31 is slot 25. Node A remains sleep in slot 25 and node B remains sleep in slots 23, 24, 28, and 31. Node C still works according to the original Disco. When node D becomes the neighbor of nodes A, B, and C after slot 20, D and B discover each other in slot 25, D and C discover each other in slot 26. B can carry the information that A is very close to B in its broadcast message. Then, when D receives the broadcast message, D also regards A as its neighbor according to the principle of neighbors’ spatial similarity [9,10]. In the information sharing step (as stated in Section 4) of the group, B will tell A that D is also A’s neighbor.

In summary, this simple example shows that D discovers A, B, and C and A, B, and C also discover D before slot 31 in the VN-NDP method. In contrast, in EQS, node D fails to discover A, B, and C; A, B, and C also fail to discover node D. EQS method filters out a part of time slots, although the energy consumption will be reduced, the neighbor discovery rate will also decrease correspondingly. Therefore, our method, VN-NDP, is aimed at overcoming the limitation of EQS and balancing energy consumption with a neighbor discovery rate.

## 4. Method Design

Firstly, the distance between two nodes A and B, $d_{AB}$, is obtained based on the RSSI signals received like in [37], and is given as follows:

(1) $$d_{AB}=10^{\frac{RSSI_{AB}-RSSI_{max}}{10m}},$$

where $RSSI_{max}$ is the maximum received signal power; *m* is path loss exponent.

Our proposed method, VN-NDP, is an indirect neighbor discovery method that works with a pairwise method, i.e., Disco. Nodes can form a group if the distance between them is less than a small value. (Note that this small threshold value has an impact on discovery performance and depends on a specific scenario. Based on many experiments, this value is set to 20 m in this paper to obtain good discovery performance and energy-efficiency.)All nodes in a group will not work according to their original wake-up schedule. They only remain active in a fraction of active slots in the wake-up schedule of one of these nodes, and the combination of these nodes’ active slots forms the whole active slots of a node that only uses the pairwise method. Therefore, nodes outside this group can regard it as a virtual node. Assume that there are *n* nodes in group *g* and that the energy consumption rate of a node is *r* when the node works according to a pairwise method, such as Disco. In addition, assume that the time when the nodes in group *g* work as a virtual node is $T_{2}$. Since group *g* works only according to one node’s wake-up schedule, the energy consumption within time $T_{2}$ is $rT_{2}$. However, since the pairwise method is used, the energy consumption for the *n* nodes within time $T_{2}$ is $nrT_{2}$. As a result, energy consumption within time $T_{2}$ is reduced by $\left(n-1\right)rT_{2}$ if VN-NDP is employed. Although it is highly energy-efficient to let nodes in one group work as a virtual node (we call this step group cooperating step), it is very challenging to manage a cooperating group throughout its lifetime. Firstly, it is challenging to establish a group (we call this step group establishing step) in an optimal energy-efficient manner in low duty cycle (LDC) mobile WSNs. In mobile WSNs, when any two nodes have discovered each other but have not joined a group, they can establish a new group if their distance is not more than the given distance threshold value. After the group is formed, other nodes can continue to join the group. However, the nodes that previously joined the group do not know whether there are new nodes that have joined the group after them. An energy-efficient manner means that the node that has joined the group tells its discovered neighbor the timestamp of the information exchange step, and all joined nodes can wake up simultaneously in the information exchange step and share their local information about group members. After sharing information, each node has the complete group information. In the sharing information step, all nodes in the group work as a virtual node and their wake-up schedules are re-planed according to the wake-up schedule of one node in the group.

From the statement above, we know that the time of the group establishing step, i.e., $T_{1}$, must be given at first. Similarly, the time of the group cooperating step, i.e., $T_{2}$, must be given in advance. The values of $T_{1}$ and $T_{2}$ greatly impact the energy-efficiency performance. The theoretical analysis of $T_{1}$ and $T_{2}$ and their optimal values are given in the next section. The remainder of this section presents the four steps of group management throughout its lifetime and gives an example to detail the design of our proposed method, VN-NDP. These four steps are group establishing step, group information sharing step 1, group cooperating step, and group information sharing step 2. The description about re-planning the wake-up schedule of group members to make them work as virtual nodes is contained in group information sharing step 1. Group dismissing operation is contained in group information sharing step 2. The details of these four steps are stated subsequently.

*Group Establishing Step:*

**Figure 3:**
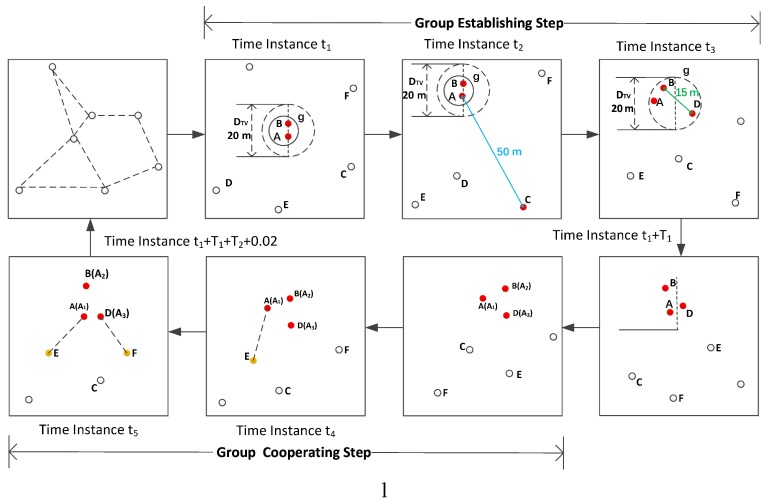
An example of our proposed method VN-NDP.

When any two nodes that have not joined a group discover each other, they establish a new group if their distance is not more than the given threshold value, i.e., $D_{TV}$. If a member node in the group establishing step discovers a new neighbor that has not joined a group and their distance does not exceed $D_{TV}$, this new neighbor can join the group. In the group establishing step, the member node informs the newly joined member the end time of the step. After this step, all nodes in the group wake-up simultaneously to share their local group information. For example, node A and node B discover each other at time instance $t_{1}$ (see Figure 3). They establish a new group *g* because they meet the condition of group establishment: (1) their distance is not more than $D_{TV}$ = 20 m; (2) they have not joined any group or their group(s) have been dismissed before $t_{1}$. Node A discovers node C at time instance $t_{2}$. Since the distance between A and C is about 50 m, which is more than $D_{TV}$ = 20 m, C cannot join group *g*. Node B discovers node D at time instance $t_{3}$. Since node B is in group *g*, we can say that group *g* discovers D. Since the distance between B and D is about 15 m, which is less than $D_{TV}$ = 20 m, D joins group *g* at time instance $t_{3}$. In addition, node B informs node D that the end time of the group establishing step is $t_{1}+T_{1}$, where $T_{1}$ is the duration of this step. Then, A, B, and D will wake up at time instance $t_{1}+T_{1}$, and they all come into the group information sharing step 1 at time instance $t_{1}+T_{1}$.

*Group Information Sharing Step 1:*

There are three sub-steps in this step. One is sharing the local group information of each member node in the group, and this information is integrated into the complete group information. In this sub-step, if the distance between any two nodes A and B, d(A,B) is more than the value of $D_{TV}$, one of the two nodes adds into this group, and the other is removed from this group. If $g_{size}\left(A\right)<g_{size}\left(B\right)$, node B is removed; otherwise, node A is removed if $d\left(A,B\right)>D_{TV}$, where $g_{size}\left(A\right)$ is the value of group size when A is in the group but the other of the two nodes, B, is not in the group. Note that only a few of the nodes are removed, since nodes generally are uniformly deployed.

The group enters another sub-step: re-planning the wake-up schedules of group members to let them work as a virtual node. Specifically, these nodes remain sleep except for the slots which are for the purpose of sharing responsibility for activating tasks of one member’s wake-up schedule. In other words, the combination of these nodes’ wake-up schedules forms a complete wake-up schedule of one node that only uses one pairwise method. In the example given in Figure 3, the wake-up schedules of group member nodes A, B, and D are re-planned in this sub-step. Since our proposed method, VN-NDP, is an indirect neighbor discovery method that works with a pairwise method, the DCs of the nodes in the group are the same. We can randomly select a node’s wake-up schedule as the working schedule of the virtual group. In this example, node A is selected. From the viewpoint of nodes outside the group, in the group cooperating step, these three nodes work together as node A, that is, they share the activating task of node A and remain sleep in other slots. This sub-step outputs the re-planned result of wake-up schedules of all member nodes. The result contains a set of tuples, and each tuple contains three elements, namely node ID, ID of an active slot whose working state is re-planned (or changed), and working state of the active slot after re-planning. In the third sub-step, all member nodes are informed of the end time of the group cooperating step. After this sub-step, all member nodes enter the next step.

*Group Cooperating Step:*

In this step, all member nodes work according to the results of the wake-up schedules re-planned in the second sub-step of step 2. In the group cooperating step, all nodes can discover new neighbors. The new neighbors discovered by members can be shared among all members in the group information sharing step 2. A discovered neighbor also can regard all group members in the group as its neighbors. The duration of this step is $T_{2}$. When the end time of the group cooperating step is reached, i.e., at $t_{1}+T_{1}+T_{2}+0.01$ seconds (the duration of the group information sharing step 1 is one slot, namely, 0.01 s), all member nodes enter the group information sharing step 2. In the given example in Figure 3, node A discovers node E at time instance $t_{4}$, and node D discovers node F at time instance $t_{5}$.

*Group Information Sharing Step 2:*

This step contains two sub-steps. The first one is that each member node shares other nodes’ discovered neighbors. In the example given in Figure 3, after this sub-step, the new neighbors E and F (discovered by A and D) are regarded as the neighbors of A, B, and D. The second sub-step is to dismiss the group. After this sub-step, all member nodes, such as A, B, and D in the example, are removed from the group, and the status of this group becomes invalid. However, these nodes can continue to join other groups or establish a new group based on these four steps in future.

The above group management process is shown in Algorithm 1. It is worth noting that, during the process from virtual group establishment to dissolution, all nodes are moving dynamically, that is, the virtual group is dynamic. The obtained neighborship is probabilistic in mobile networks [15], and we ensure that the probability value of neighborship is larger in group cooperating steps by selecting appropriate values of the group size $size_{g}\left(ge\right)$ at the end of group establishing step and the group size $size_{g}\left(gc\right)$ at the end of group cooperating step $size_{g}\left(gc\right)$ is decided by $size_{g}\left(ge\right)$ and $T_{2}$). When the group dissolves, or some nodes move too far beyond the group threshold, these nodes will resume their own wake-up schedules and wait for the next group opportunity in the process of continuous neighbor discovery.

**Algorithm 1:** Group management process.

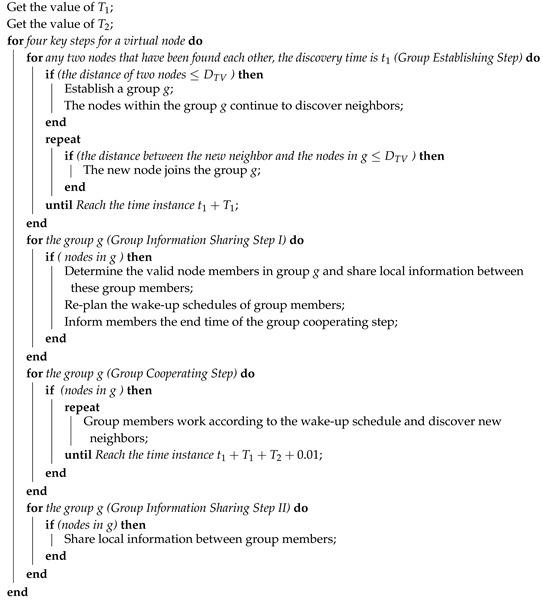



## 5. Theoretical Analysis

Suppose that the number of nodes in a group is *n*. The larger the *n* is, the greater the group size is (assuming that the nodes are evenly distributed, the more nodes there are in the group, the larger the group area is) and the smaller the value of $T_{2}$. The contradiction is: on the one hand, the smaller the value of $T_{2}$, the number of times the group turns into group information sharing step 1 increases, and the more energy consumption there is; on the other hand, the larger *n* is, the less energy each node consumes in group cooperating steps. Therefore, there is an optimal value of *n*, $n_{opt}$. Our objective in this section is to obtain $n_{opt}$ to minimize the value of EC, where EC is the energy consumption in the network per second. After $n_{opt}$ is obtained, the value of $T_{2}$ can be obtained based on the quantitative relationship between $T_{2}$ and $n_{opt}$ obtained by the method mentioned below. To let the node number at the end of group establishing step be as close as possible to $n_{opt}$, the value of $T_{1}$ is also deduced at the end of this section.

Since all nodes work according to the pairwise method used in the group establishing step, we only pay close attention to the energy consumption in the group information sharing step 1, group cooperating step, and group information sharing step 2. The time of the group information sharing step 1 and group information sharing step 2 is one slot, respectively, and the duration is assumed to 10 milliseconds. Then, the total time of these three steps is $t_{total}=T_{2}+0.02$ seconds. Assume that the energy consumption in one slot is one unit and that the DC of each node that works according to the pairwise method used is *x*. Then, the energy consumption per second for one node that works according to the pairwise method used is 100x. Assume that there are *n* nodes in a group. Since the group works as a node, i.e., a virtual node, the energy consumption per second of the group is 100x in the group cooperating step. Therefore, the energy consumption per second for each node in the group cooperating step is $\frac{100x}{n}$. In addition, there are two slots in the group information sharing step 1 and group information sharing step 2 within the time of $t_{total}$. The energy consumption used for information sharing per second is $\frac{2}{t_{total}}$. Finally,

(2) $$EC=\frac{2}{T_{2}+0.02}+\frac{100x}{n}.$$

From Equation (2), we can see that the values of $T_{2}$ and *n* greatly impact EC. Our objective is to minimize EC by selecting appropriate values of $T_{2}$ and *n*. After that, we can get the optimal $T_{1}$ to make the number of group members be approximately equal to the appropriate *n*.

Next, we discuss how to obtain the expression $T_{2}$. In the group information sharing step 1, each member node can obtain the complete information of this group, including group size and number of member nodes. Then, after the group information sharing step 1, the expected group size change with time can be obtained based on the group size, the number of member nodes in the group information sharing step 1, node movement speed, and node movement model. We use the maximum distance between any two member nodes in the group to denote the group size. Group size has a great impact on the performance of neighbor discovery since a very large group size causes many wrongly discovered neighbors. Therefore, the group size must be small. To achieve this goal, the expected group size must not be more than a given threshold value, i.e., $d_{TV2}$. Note that the value of $d_{TV2}$ is application-dependent, and all groups in a network use the same given $d_{TV2}$. Assume that the group size in the group information sharing step 1 is $d_{0}$, and the expected group size change over time is *d*. Then, *d* is a function of $d_{0}$, the number of member nodes *n*, and time length after the group information sharing step 1 if the node movement model is given and the movement speed of each node remains the same and unchanged, that is, $d=f(d_{0},n,t)$. Then, $d_{TV2}=f\left(d_{0},n,T_{2}\right)$. If the expression $f(d_{0},n,t)$ can be obtained, the value of $T_{2}$ can be gotten. Next, we discuss how to obtain the expression $f(d_{0},n,t)$. Generally, it is very hard to directly obtain the accurate estimation, since the used movement model is probabilistic. In this paper, by using a specific movement model, we capture the evaluated value of distance increment through a test method based on a large number of simulation experiments. Our simulations indicate that, when the experiment time is large enough, the value of *d* is approximated to a fixed value when node speed *s* is given in advance. In this paper, we employ the node movement model used in many existing neighbor discovery methods [9,10,15,21]. Note that, for other node movement models, we can obtain the value of distance increment in a similar way. The simulation scenario used in this paper (in this section and the evaluation section) is described in the evaluation section. We select one $d_{0}$ from 10, 15, 20, 25, 30 meters, one time interval from 200, 400, …, 2000 slots, and one value of *n* from 2, 3, …, 15 each time to evaluate the corresponding distance increment. Applying curve fitting to the result data, if node moving speed *s* is 1 m/s, *d* can be expressed as a function of $d_{0}$, *n* and *t* as follows:

(3) $$d=c_{0}+c_{1}n+c_{2}d_{0}+c_{3}t+c_{4}nd_{0}+c_{5}nt+c_{6}d_{0}t,$$

where $c_{0}$ = 13.251, $c_{1}$ = 1.453, $c_{2}$ = 0.317, $c_{3}$ = 0.42, $c_{4}$ = 0.137, $c_{5}$ = 0, $c_{6}$ = 0. Let $d=d_{TV2}$, then,

(4) $$T_{2}=\frac{d_{TV2}-c_{0}-c_{1}n-c_{2}d_{0}}{c_{3}+c_{4}n},$$

where only *n* is an independent variable, $d_{0}$ is a constant because its value is known in the group information sharing step 1. Substituting Equation (4) into Equation (2), we get the following:

(5) $$EC=\frac{2(c_{3}+c_{4}n)}{d_{TV2}-c_{0}-c_{1}n-c_{2}d_{0}+0.02\left(c_{3}+c_{4}n\right)}+\frac{100x}{n}.$$

Taking the derivative with respect to *n*, the optimal value of *n* is as follows:

(6) $$n_{opt}=\frac{-4801.30x+0.47\beta -114.93d_{0}x+362.57d_{TV2}x+0.01.6d_{0}\beta -0.03d_{TV2}\beta )}{0.22d_{0}-0.69d_{TV2}+52.58x+6.03},$$

where $\beta =\sqrt{x(-43429d_{0}+137000d_{TV2}-1205127)}$. Sometimes, $n_{opt}$ is not an integer. In this case, it can be rounded up to the nearest positive integer. After obtaining the value of $n_{opt}$, the optimal value of $T_{2}$ and the minimum value of EC(n) can be obtained.

The main objective of the group establishing step is to ensure that the number of nodes in a group is close to $n_{opt}$ as possible. Assume that the expected time for a member node to discover another member node is $t_{\epsilon }$. Next, we will deduce that the time of the group establishing step can be expressed by $t_{\epsilon }$ and $n_{opt}$. Finally, the calculating process of the value of $t_{\epsilon }$ according to known parameters is presented. At the beginning of the group establishing step, there are two member nodes in a group, denoted by nodes A and B. After $t_{\epsilon }$, A and B discover a member node each, namely, C and D. After $2t_{\epsilon }$, A and B discover a member node each, namely E and F, and C and D discover a member node each, namely, G and H. After 0, $t_{\epsilon }$, $2t_{\epsilon }$, …, $kt_{\epsilon }$, the numbers of member nodes are 2, 4, 8, …, $2^{k+1}$ respectively. If $n_{opt}$ is just equal to $2^{k+1}$, $k=log_{2}n_{opt}-1$. Then, the time of the group establishing step, i.e., $T_{1}$, is given as follows:

(7) $$T_{1}=\left(log_{2}n_{opt}-1\right)t_{\epsilon }.$$

If $n_{opt}$ can be divided by $2^{k+1}$ with remainder, $T_{1}$ is given as follows:

(8) $$T_{1}=\left(\left\lfloor log_{2}\left(n_{opt}\right)\right\rfloor -1\right)t_{\epsilon }+\frac{n_{opt}-2^{\left\lfloor log_{2}n_{opt}\right\rfloor }}{2^{\lfloor log_{2}n_{opt}\rfloor -1}}t_{\epsilon }.$$

Next, we deduce the expression of $t_{\epsilon }$. Assume that the expected latency for any two nodes to discover each other is *l* and that the node density of the network is ρ. Then, the number of nodes in the group area is given as follows:

(9) $$N_{g}=\rho \frac{\pi d_{TV1}^{2}}{\pi r^{2}}=\rho \frac{d_{TV1}^{2}}{r^{2}}.$$

Then,

(10) $$t_{\epsilon }=\frac{l}{N_{g}}=\frac{l}{\rho \frac{d_{TV1}^{2}}{r^{2}}}=\frac{lr^{2}}{d_{TV1}^{2}}.$$

## 6. Performance Evaluation

The simulation scenario used in this paper is described as follows: The nodes are deployed in a 500×500 network area that is divided into equal-sized grids whose side lengths are all 10 m. In other words, there are 2500 equal-sized grids in the network area. The node movement model used in our evaluation was also used in many other studies [9,10,15,21]. The movement model is that nodes are deployed at the edges of the grids and move on the edges. When a node arrives at a vertex of a grid, the node randomly selects one direction from left, right, up, down, and continues to move.

In our simulation, we set the slot length to be 25 ms like ACC [16] and GBFA [9], rather than a 10 ms like direct method [25], for two reasons: (1) a too-small slot will lead to a collaborative wake-up schedule not being able to be realized properly because of the clock jitters; (2) for indirect discovery, a bigger slot will reduce collisions of messages and enable more exchanging packets used to negotiate the joint schedule and exchange neighbor tables. (Note that enlarging slot length will not lead to the changes of DC and energy-efficiency, and exchanging more packets in a slot will also not lead to much energy consumption, since the node almost consumes the same energy in sending and listening states.)

We compare our proposed method, VN-NDP, with other methods in three cases. Firstly, we compare VN-NDP with the indirect discovery method EQS and the direct discovery method Disco. In this case, for comparison with the direct discovery method Disco, both VN-NDP and EQS use Disco as their underlying neighbor discovery method. Secondly, we compare VN-NDP with the indirect discovery method EQS and the direct discovery method Searchlight. In this case, for comparison with the direct discovery method Searchlight, both VN-NDP and EQS use Searchlight as their underlying neighbor discovery method. In addition, in Section 6.4 we compare VN-NDP with the indirect discovery method EQS when the direct discovery method used is a slot-less method Griassdi [30]. The communication radius of each node is set to 100 m. We compare the average discovery latencies (ADLs) and discovered neighbor percentages (DNPs) of all algorithms when the total energy budget of all algorithms, i.e., the total DC, remained equal for fair comparisons. ADL is obtained via dividing the total discovery latencies by the total discovered neighbors of all nodes.

DNP is obtained via dividing the total discovered neighbors by the total geographical neighbors of all nodes. The discovered neighbor will be invalid after a period time $T_{valid}$ if there is no new discovery between the two nodes within $T_{valid}$. The value of $T_{valid}$ is obtained by the expected time when the two nodes are not neighbors. This expected time is the function of the distance between the two nodes when they discover each other, node moving model, and node speed, and we use the curve-fitting method stated in Section 5 to get the expression of this function. Note that, although the discovered neighbor sometimes has become invalid before $T_{valid}$, it is proper to use this expected time to obtain DNP. The reason is that the obtained neighborship is probabilistic in mobile networks [15], and the probability value of neighborship is larger before $T_{valid}$.

We say a node B is the geographical neighbor of another node *A* if B is in the communication area of A, whether A receives B’s RF signals or not. DNP is similar to CDF (cumulative distribution function), but, in mobile networks, DNP is more proper than CDF because the geographical neighbor and discovered neighbors are time-varying. Under the same simulation parameters, the smaller the ADL and the larger the DNP, the better the performance of the algorithm.

The ADLs and DNPs of all methods are evaluated and compared under the influence of total DC, node velocity, and node density. When total DC changes from 1% to 5%, node speed and node density remain constant and are set to their default values. When node speed changes from 1 m/s to 5 m/s, total DC and node density remain constant and are set to their default values. When node density changes from 30 to 50, total DC and node speed remain constant and are set to their default values. The default values of total DC, node speed, and node density are 3%, 1 m/s, and 35 respectively.

### 6.1. Impact of DC

We set the movement speed of nodes to 1 m/s. The number of nodes in the network area is 280, that is, the node density is 35. As a variable, the DC changes from 1% to 5%. Under these conditions, the ADLs and DNPs of the three neighbor discovery methods are compared.

**Figure 4:**
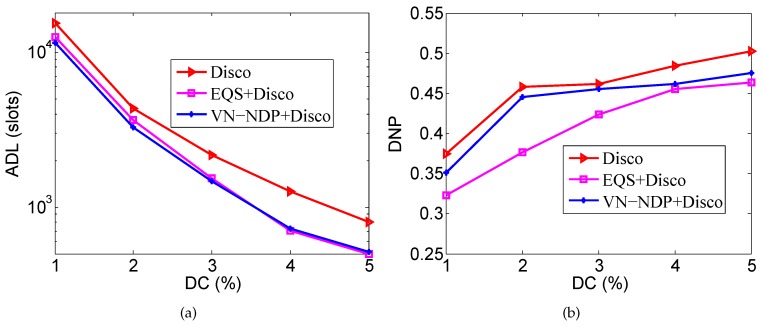
Impact of DC when the pairwise method is Disco. (**a**) shows the effect of DC on the ADLs and (**b**) shows the effect of DC on the DNPs of the three algorithms when the pairwise method is Disco. DC: duty cycle.

Figure 4a,b respectively show the effect of DC on the ADLs and DNPs of the three algorithms when the pairwise method is Disco. In Figure 4a, the ADLs of EQS+Disco and VN-NDP+Disco are much lower compared with the Disco method because EQS+Disco and VN-NDP+Disco both efficiently re-plan the original discovery wake-up schedules by using the information of discovered neighbors to increase the average discovery delay. When DC is less than or equal to 3%, the ADL of VN-NDP+Disco is always less compared with EQS+Disco, indicating that VN-NDP+Disco is better than EQS+Disco in terms of ADL performance in this case. For example, when the DC is equal to 2%, the ADLs of VN-NDP+Disco and EQS+Disco are 3282 and 3648, respectively, and VN-NDP+Disco reduces the ADL by about 10.03% compared with EQS+Disco. When the DC is greater than or equal to 4%, the ADL of VN-NDP+Disco is slightly larger compared with EQS+Disco, and the ADL performance of VN-NDP+Disco is slightly worse under this condition. The main reason may be that, when the DC is large, the amount of group information sharing in the VN-NDP method increases, thereby consuming more energy. In Figure 4b, the DNPs of these methods tend to increase with a change in DC. In the case of a lower DC, there is a higher possibility that a neighbor discovered by a node at a certain time may move physically beyond the neighbor range, that is, the two nodes are no longer geographical neighbors. As the DC changes, the DNPs gradually increase. Since both EQS and VN-NDP remove some active slots more or less, the overall DNPs are lower compared with Disco. However, our proposed method guarantees the integrality of nodes’ wake-up schedules, so, in terms of DNP, our method performs better than EQS. For instance, when the DC is 2%, the DNP of EQS+Disco is 0.376, and that of VN-NDP+Disco is 0.445. Our method improves the DNP by 18.35% compared with the EQS+Disco method.

**Figure 5:**
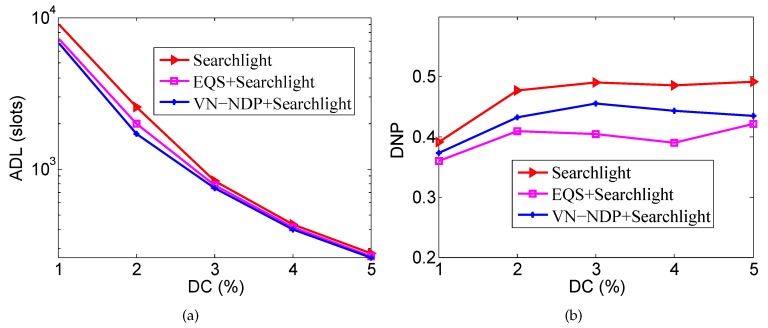
Impact of DC when the pairwise method is Searchlight. (**a**) shows the effect of DC on the ADLs and (**b**) shows the effect of DC on the DNPs of the three algorithms when the pairwise method is Searchlight.

Figure 5a,b respectively show the effect of DC on the ADLs and DNPs of the three algorithms when the pairwise method is Searchlight. Similar to Figure 4a, Figure 5a shows that the ADLs of EQS+Searchlight and VN-NDP+Searchlight are much lower compared with the Searchlight method. The ADL of VN-NDP+Searchlight is always less than EQS+Searchlight with a change in DC, indicating that VN-NDP+Searchlight is better than EQS+Searchlight in terms of ADL performance. For example, when the DC is equal to 2%, the ADL of VN-NDP+Searchlight and EQS+Searchlight are 1716 and 1994, respectively; the ADL of VN-NDP+Searchlight is reduced by 13.94% compared with EQS+Searchlight. Similar to Figure 4b, in Figure 5b, the DNPs of these methods tend to increase with a change in DC. The DNP of VN-NDP+Searchlight is always much larger than that of EQS+Searchlight, showing that the DNP performance of VN-NDP+Searchlight is always much better than that of EQS+Searchlight. For instance, when the DC is 2%, the DNP of EQS+Searchlight is 0.41 and that of VN-NDP+Searchlight is 0.433. Our method improves the DNP by 5.61%.

### 6.2. Impact of Node Density

We let the nodes’ movement speed be 1 m/s and the DC be 3%. The node number is a variable; starting from the initial 280 nodes, adding 40 nodes at a time up to 400 nodes, that is, the node density ranges from 30 to 50. Under these conditions, we explore the changes in the ADLs and DNPs of all the methods.

**Figure 6:**
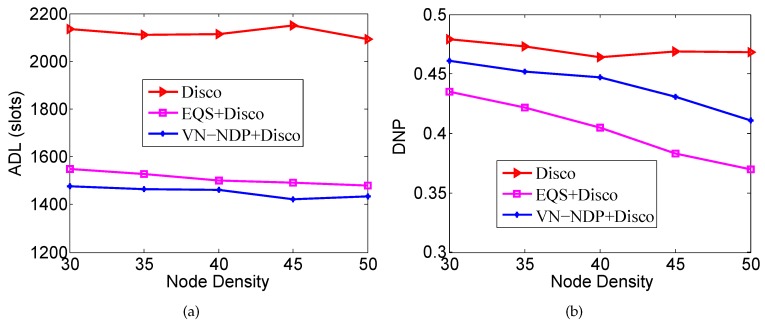
Impact of node density when the pairwise method is Disco. (**a**) shows the effect of node density on the ADLs and (**b**) shows the effect of node density on the DNPs of the three algorithms when the pairwise method is Disco.

Figure 6a,b show the effect of node density on the ADLs and DNPs of the three algorithms when the pairwise method of VN-NDP and EQS is Disco. As shown in Figure 6b, the ADLs of VN-NDP+Disco and EQS+Disco are always much smaller than the ADL of Disco, while the ADL of VN-NDP+Disco is always smaller than the ADL of EQS+Disco. For example, when the node density is 50, the ADL of VN-NDP+Disco, EQS+Disco, and Disco are 1435, 1480, and 2094, respectively. The ADLs of VN-NDP+Disco and EQS+Disco are 68.53% and 70.68% of that of Disco, respectively. As the node density increases, the ADL of Disco is almost unchanged, while those of VN-NDP+Disco and EQS+Disco decrease slightly. With the increase of node density, VN-NDP+Disco and EQS+Disco can utilize more neighbor information more efficiently to re-plan the original discovery wake-up schedules. As shown in Figure 6b, the DNP of Disco is hardly affected by the node density. The DNPs of VN-NDP+Disco and EQS+Disco decrease as the node density increases. This is because VN-NDP+Disco and EQS+Disco both destroy the original discovery wake-up schedules to different degrees. The negative effects of the non-integrity on DNP increase as the node density increases. However, the DNP of VN-NDP+Disco is always greater than that of EQS+Disco, indicating that the DNP performance of VN-NDP+Disco is always better than that of EQS+Disco with change in node density.

**Figure 7:**
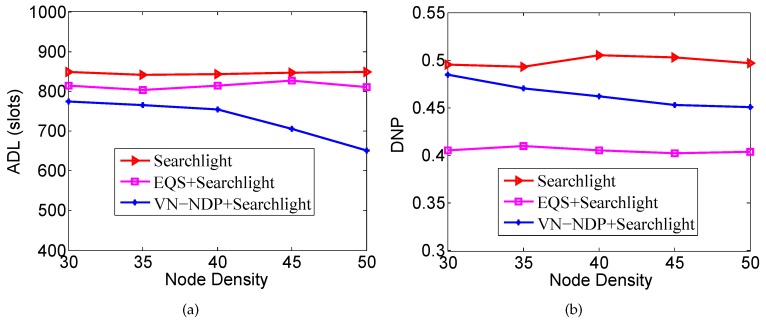
Impact of node density when the pairwise method is Searchlight. (**a**) shows the effect of node density on the ADLs and (**b**) shows the effect of node density on the DNPs of the three algorithms when the pairwise method is Searchlight.

Figure 7a,b show the effect of node density on the ADLs and DNPs of the three algorithms when the pairwise method of VN-NDP and EQS is Searchlight. When the pairwise method is Searchlight, the ADL and DNP performances of the three algorithms are similar to their performances when the pairwise method is Disco as the node density changes. In Figure 7a, Searchlight’s ADL is hardly affected by node density, while VN-NDP+Searchlight and EQS+Searchlight’s ADLs generally decrease with node density; VN-NDP+Searchlight’s ADL is always less than that of EQS+Searchlight. In the process of node density change, the ADL of VN-NDP+Searchlight is reduced by up to 19.73% compared with EQS+Searchlight. In Figure 7b, the DNP of Searchlight is hardly affected by the node density, while the DNPs of VN-NDP+Searchlight and EQS+Searchlight generally decrease as the density of nodes increases. In this change process, the DNP of VN-NDP+Searchlight is increased by up to 19.75% compared with EQS+Searchlight.

### 6.3. Impact of Speed

In this scenario, the node density is 280 and the DC is 3%. As the variable, we start the nodes’ speed from 1 m/s, and it is increased by 1 m/s each time until it reaches 5 m/s. We compare the ADLs and DNPs of all the methods mentioned.

**Figure 8:**
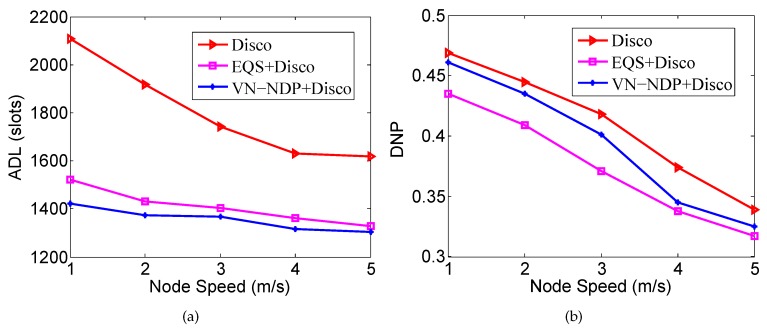
Impact of node speed when the pairwise method is Disco. (**a**) shows the effect of node movement speed on the ADLs and (**b**) shows the effect of node movement speed on the DNPs of the three algorithms when the pairwise method is Disco.

Figure 8a,b show the effect of node movement speed on the ADLs and DNPs of the three algorithms when the pairwise method of VN-NDP and EQS is Disco. Since a higher speed indicates a shorter average time for two nodes to encounter each other and depart, the ADLs of all methods decrease with increasing node speed. It is worth mentioning that, among all the algorithms, the ADL of our VN-NDP is the lowest at different node movement speeds (see Figure 8a). For example, when the node speed is 4 m/s, the ADL of VN-NDP+Disco is only 1317, and the ADLs of Disco and EQS+Disco are 1362 and 1631, respectively. Compared with EQS+Disco, the ADL of VN-NDP+Disco is reduced by about 3.42%. In Figure 8b, as the movement speed of nodes increases, the DNPs of all methods show a downward trend. The DNPs of VN-NDP+Disco and EQS+Disco are relatively lower than that of the Disco algorithm. Nevertheless, it is obvious that our method always performs well relative to EQS+Disco. For example, when the node speed is 3 m/s, the DNPs of VN-NDP+Disco and EQS+Disco are 0.401 and 0.371, respectively. Compared with EQS+Disco, the DNP of VN-NDP+Disco is increased by about 8.09%.

**Figure 9:**
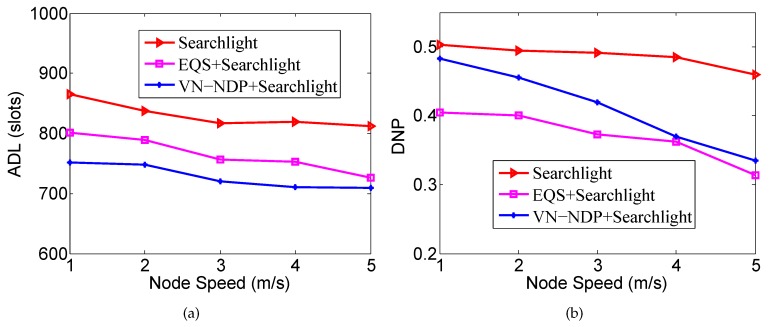
Impact of node speed when the pairwise method is Searchlight. (**a**) shows the effect of node movement speed on the ADLs and (**b**) shows the effect of node movement speed on the DNPs of the three algorithms when the pairwise method is Searchlight.

Figure 9a,b show the effect of nodes’ movement speed on the ADLs and DNPs of the three algorithms when the pairwise method of VN-NDP and EQS is Searchlight. Similar to Figure 8a, in Figure 9a, the ADLs of these three methods all decrease with increase in node speed, and the ADL of VN-NDP+Searchlight is always less than those of EQS+Searchlight and Searchlight. For example, when the node speed is 3 m/s, the ADL of VN-NDP+Searchlight is only 720, while the ADLs of EQS+Searchlight and Searchlight are 756 and 817, respectively. On the other hand, similar to Figure 8b, in the comparison of DNPs, as the movement speed of nodes increases, the DNPs of all the methods show a downward trend. For example, in Figure 9b, when nodes’ movement speed is 3 m/s, the DNPs of VN-NDP+Searchlight, EQS+Searchlight, and Searchlight are 0.419, 0.373, and 0.491, respectively.

### 6.4. Impact of Speed When the Pairwise Method Is Griassdi

In this scenario, the node density is 280, the DC is 3%, and the pairwise method is Griassdi. As the variable, we start the nodes’ speed from 1 m/s, and it is increased by 1 m/s each time until it reaches 5 m/s. We compare the ADLs and DNPs of all the methods mentioned.

**Figure 10:**
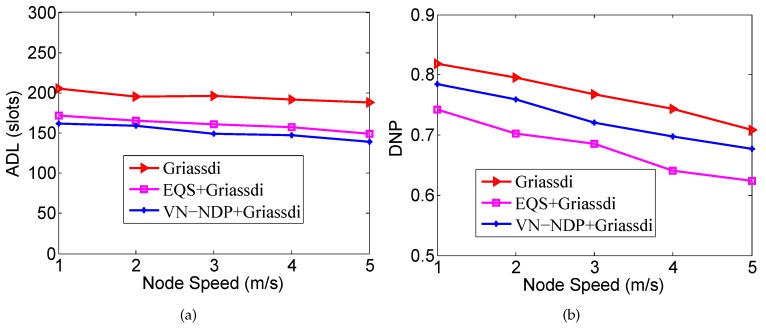
Impact of node speed when the pairwise method is Griassdi. (**a**) shows the effect of node movement speed on the ADLs and (**b**) shows the effect of node movement speed on the DNPs of the three algorithms when the pairwise method is Griassdi.

Figure 10a,b show the effect of node movement speed on the ADLs and DNPs of the three algorithms when the pairwise method of VN-NDP and EQS is Griassdi. Among all the algorithms, the ADL of our VN-NDP is the lowest at different node movement speeds (see Figure 10a). For example, when the node speed is 3 m/s, the ADL of VN-NDP+Griassdi is only 149, and the ADLs of Griassdi and EQS+Griassdi are 196 and 161, respectively. Compared with EQS+Griassdi, the ADL of VN-NDP+Griassdi is reduced by about 7.45%. In Figure 10b, as the movement speed of nodes increases, the DNPs of all methods show a downward trend. The DNPs of VN-NDP+Griassdi and EQS+Griassdi are relatively lower than that of the Griassdi algorithm. Nevertheless, it is obvious that our method always performs well relative to EQS+Griassdi. For example, when the node speed is 2 m/s, the DNPs of VN-NDP+Griassdi and EQS+Griassdi are 0.759 and 0.703, respectively. Compared with EQS+Griassdi, the DNP of VN-NDP+Griassdi is increased by about 7.97%.

## 7. Discussion

Neighbor discovery protocol plays an important role in wireless sensor networks because it is a prerequisite for communication between nodes in WSNs. Due to the limited energy of sensor nodes, improving the energy efficiency of processes in WSNs is the key to extending the network lifetime. The neighbor discovery protocol also needs, without exception, improvement in energy efficiency as much as possible and at the same time to ensure that the discovery delay is as small as possible. In recent years, a number of efficient indirect neighbor discovery protocols have been proposed to further improve neighbor discovery performance. These indirect methods are generally divided into two categories: the indirect methods of adding some active time slots and the indirect methods of removing some active time slots. The VN-NDP method proposed in this paper combines the advantages of the above two methods; hence, the performance of neighbor discovery is greatly improved. In order to achieve this goal, the VN-NDP method proposes the combination of multiple neighboring nodes into a cooperative group, and the group is treated as a virtual node so as to maintain the integrity of the original wake-up schedules as much as possible. VN-NDP thereby overcomes the limitation of the EQS method, that is, removal of existing active time slots results in a greatly reduced possibility of discovering new neighbors. On this basis, this paper proposes a set of management mechanisms throughout the lifetime of the group, including group establishment, group coordination, and group dissolving operations. The energy efficiency and duration of each stage of the group are theoretically analyzed to select optimal parameters. Simulation experiments in multiple scenarios verify the effectiveness of the proposed method.

However, there are some limitations in our proposed method VN-NDP. The first limitation of VN-NDP is that only simulations are performed. In the future, we will establish a test-bed to verify the simulations. The second main limitation of VN-NDP is that, in the process of intra-group node synchronization or sharing of information between nodes in a group, all nodes in the group are required to wake up in a certain global time slot. This method wastes more energy and affects the further improvement of VN-NDP’s energy efficiency. In the future, we will explore a more efficient collaborative group management mechanism to improve the energy-efficiency of neighbor discovery protocol based on virtual nodes. In addition, through the experimental simulation results, it is found that re-planning the wake-up time slots has a great impact on the neighbor discovery performance, including the average discovery delay and the neighbor discovery probability. How to make full use of the information of the neighbors that have been discovered to more reasonably design or re-plan the neighbor discovery wake-up schedule and to propose a more energy-efficient indirect neighbor discovery protocol is also our main research direction in the future.
